# A System of Medicine

**Published:** 1898-03

**Authors:** 


**A System of Medicine.** By Many Writers. Edited by THOMAS CLIFFORD ALLBUTT, M.D., F.R.S. Vol. IV. Pp. xii., 880. London: Macmillan and Co., Limited. 1897.

Over two hundred and fifty pages of this volume are occupied with Diseases of the Liver: of these, Dr. William Hunter is responsible for more than a hundred, and his name is a sufficient guarantee that the present knowledge of the physiology and pathology of the largest gland in the body is well represented. He shows that jaundice is always the result of absorption, that it is caused by changes in the liver and in the bile, and is thus in every sense hepatogenous, the result of absorption of bile formed and excreted by the liver. "The cause of the absorption may be obvious—mechanical obstruction (*Simple hepatogenous jaundice*), or more obscure and less easily demonstrable swelling and catarrh of the lining epithelium of the bile passages, with consequent increased viscidity of the bile (*Hæmo-hepatogenous jaundice*)." The influence of catarrhal conditions of the mucous membrane is also manifest in the production of cholesterin and cholelithiasis; for we find that an "increased formation of cholesterin in connection with subacute inflammatory and catarrhal conditions of the lining membrane of the bile passages, especially of the gall-bladder, is the chief factor underlying the formation of gall-stones in disease. The cholesterin which goes to form gall-stones has never been in solution in the bile. It is formed as viscous material within the degenerated epithelium thrown off from the gall-bladder." Hence the conclusion follows: "that the large group of morbid conditions comprised under the term cholelithiasis are due primarily to disorder of the bile passages, not to functional disorder of the liver." Suppurative Hepatitis is described by Dr. Andrew Davidson, of Mauritius; and Amœbic Abscess by Dr. Henri A. Lafleur, of Montreal. Mr. Mayo Robson is responsible for the chapter on Diseases of the Gall-bladder and Bile-ducts, and Cholangitis; and Dr. Reginald H. Fitz gives us nearly twenty pages on Diseases of the Pancreas.

The section on Diseases of the Kidneys occupies nearly two hundred pages: of these Dr. Dickinson has written over fifty pages on the Diseases of the Kidney characterised by Albuminuria; and it is needless to add that no one could have written a more concise and complete account of the varieties of Bright's disease. A lengthy chapter on the General Pathology of the Renal Functions is by Dr. Rose Bradford; and this is followed by one on Nephroptosis by Dr. Alexander Macalister, who comments especially on the occurrence of dilatation of the stomach, owing to the pressure on the duodenum caused by the displacement of the kidney. He speaks approvingly of the operation of nephrorrhaphy; but he does not endorse the view that a movable kidney is a continual menace to life. The various other surgical aspects of affections of the kidneys are described by Mr. Henry Morris, whose account of the distinctive points in the diagnosis of renal tumours makes it appear that they should not be the most difficult of abdominal enlargements to diagnose correctly.

Diseases of the Thyroid Gland have been entrusted jointly to Dr. W. M. Ord and Dr. Hector Mackenzie. We notice that they do not commit themselves to any theory as to the pathology of Graves's disease, and they do not favour the operative treatment; for if we compare the results with those of other methods of treatment, we find no striking difference except a death-rate of twelve per cent. due to the operation; and again, "We consider that operative removal of a portion of the thyroid is never justifiable in an acute case. In a chronic case we should only be disposed to recommend it where the tumour seriously interferes with the breathing, or where other methods of treatment have failed."

Dr. H. D. Rolleston has a section on Diseases of the Spleen, and another on Addison's Disease. He remarks that, "although the lesion is generally tuberculous, it is not necessarily so in all cases," and that the results of treatment of Addison's Disease by suprarenal gland-substance as compared with thyroid in myxœdema are at present disappointing.

A chapter on Hodgkin's Disease by Dr. George R. Murray is followed by one on Scrofula, the joint product of Professor Allbutt and Mr. Pridgin Teale, who advocate the method of free removal of the glands as suggested by the Editor in a paper which he read at the International Medical Congress in London in 1881.

Sir Dyce Duckworth sums up what is known on the subject of Obesity, and he strikes the correct note when he affirms "that there is nothing special or peculiar in the subject of obesity which any well-educated medical man may not be trusted to deal with. It is necessary to assert this much, because of late years this matter has been absurdly exalted into a 'speciality'—a pretension unworthy of our profession and misleading to the general public."

The three following chapters are on the diseases of the respiratory organs, the general pathology of respiratory diseases, and the treatment of asphyxia—being by Dr. A. Ransome; whilst the physical signs of the diseases of the lungs and heart are described by Dr. Hector Mackenzie.

The remaining portion of this book is on the Diseases of the Nose, Pharynx and Larynx, and is the joint product of Dr. de Havilland Hall, Dr. Greville MacDonald, Sir Felix Semon, and Dr. Watson Williams. We are glad to observe that the authors strongly condemn the common tendency to refer all and sundry obscure neuroses to the nose, and to explain their occurrence as nasal reflex phenomena; and they emphasise the importance of being on our guard against this prevalent error. They also condemn all unnecessary snipping of the uvula.

